# The influence of an all-female healthcare environment on mentorship and empowerment of female healthcare professionals

**DOI:** 10.1371/journal.pgph.0000081

**Published:** 2022-03-02

**Authors:** Naikhoba C. O. Munabi, Allyn Auslander, Meredith D. Xepoleas, Libby D. Bunker, Kella L. Vangsness, Sara Koualla, Kathy S. Magee, William P. Magee, Caroline A. Yao

**Affiliations:** 1 Division of Plastic and Reconstructive Surgery, Keck School of Medicine of USC, Los Angeles, CA, United States of America; 2 Operation Smile Inc, Virginia Beach, VA, United States of America; 3 Division of Plastic and Maxillofacial Surgery, Children’s Hospital Los Angeles, Los Angeles, CA, United States of America; 4 Faculté de Médecine et de Pharmacie d’Oujda, Oujda, Morocco; 5 Department of Plastic Surgery, Shriners Hospital for Children, Los Angeles, CA, United States of America; McGill University, CANADA

## Abstract

Low- and middle-income countries (LMICs) have the greatest need for additional healthcare providers, and women outside the workforce help address the need. Women in healthcare need more mentorship and leadership training to advance their careers due to systemic barriers. This study evaluates how women working together on a medical team influences mentorship, leadership and empowerment. A single all-female volunteer team participating in a cleft surgery mission in Oujda, Morocco were surveyed before and after the mission. Statistical analysis with student’s t-test or chi-squared were performed. 95 female volunteers from 23 countries participated on this team and 85% completed surveys. Volunteers from high-income countries (32%) and LMICs (68%) had similar mission roles (p = 0.58). Experience as a mission volunteer (p = 0.47), team leader (p = 0.28), and educator (p = 0.18) were equivalent between cohorts. 73% of women had previously received mentorship but 98% wanted more. 75% had previously mentored others, but 97% wanted to be mentors. 73% of volunteers who had no prior mentorship found their first mentor during the mission. All participants found a long-term peer relationship and felt motivated to mentor women at home. 95% were inspired to pursue leadership positions, advance professionally, and continue working with other women. This population of female healthcare professionals overwhelmingly desired more mentorship than is felt to be available. An all-female healthcare environment appears to provide opportunities for mentorship and create lasting motivation to teach, lead, and advance professionally. Findings raise the potential that increasing visibility of female professionals may effectively empower women in healthcare.

## Introduction

Eighteen million more healthcare workers, most prominently in low- and middle-income countries (LMICs), are needed globally to provide safe and accessible surgical care to the world’s population [1]. Over one billion women worldwide, the majority of whom live in LMICs, do not participate in the traditional workforce [2]. Women are therefore the largest demographic of people that can be mobilized into healthcare to fill current gaps in multiple provider roles [2].

Increasing the number of women in healthcare requires understanding barriers to entrance and advancement. While 70% of healthcare roles are currently filled by women, the majority of leadership positions are held by men [3]. Seventy-five percent of senior roles in medicine are held by men, 69% of global health organizations are led by men, and 80% of healthcare boards are exclusively men [3, 4]. Studies suggest that lack of female representation in leadership positions, restrictive cultural gender norms, and lack of mentorship contribute to limited engagement and advancement of women in healthcare [5–9].

Few studies have examined women in healthcare in LMICs, and 90% of studies on gender in surgery are from high income countries (HICs) [10]. Knowledge of baseline working conditions for women in healthcare are needed to create effective initiatives. Without engaging women in LMICs, gender equity in medicine is estimated to take over 200 years [4].

Operation Smile, a global nonprofit with approximately 6000 volunteers from over 60 countries, runs hundreds of surgical programs each year in LMICs. In the last five years, over 60% of active volunteers were women. To celebrate the contributions of women to the organization and healthcare worldwide, Operation Smile held a cleft mission with an all-female volunteer team in Oujda, Morocco on International Women’s Day 2020. With 95 female healthcare workers from 23 countries, this environment was an opportunity for women to work, learn, and establish professional relationships. This small-scale study evaluates the programmatic impact on participant experiences, mentorship, and career aspirations. We focused on whether interaction with female peers could promote mentorship, leadership development, and empowerment for women in medicine.

## Methods

### Ethics statement

Ethics approval for this study was obtained from Children’s Hospital Los Angeles (IRB #CHLA 20–00026), Operation Smile, Inc. (Virginia Beach, VA), and Operation Smile Morocco (Casablanca, Morocco). Verbal consent was obtained from all participants. All regulatory requirements to conduct this study in Morocco were met. No specific funding was received for this study.

An anonymous survey was administered during an Operation Smile cleft surgery mission in Oujda, Morocco (March 2020) with all-female team. For the purposes of this paper, female (sex) and woman (gender) were used to describe the volunteers of this study interchangeably, due to the grammar use of female as a verb. All volunteers identified as women; the authors were not focused on the biological sex of the volunteers. The following providers were surveyed: doctors (surgeons, anesthesiologists, pediatricians), nurses (operating room, intensive care/post anesthesia care unit (PACU), ward nurses), other medical volunteers (speech pathologists, dentists, child-life specialists, medical records specialists, biomedical engineers, patient imaging technicians), and nonmedical volunteers (administrators, program coordinators, translators, students). Each specialty had a team leader responsible for assignment of tasks, upholding standards of care, and multidisciplinary team communication. Volunteers present for the entire mission were eligible for participation.

The survey was developed by two focus groups, one within Operational Smile Inc and on within a pool ov volunteers independent to those participating in the mission. The survey was then evaluated by experts to ensure proper wording and structure. Lastly, the survey was distributed to volunteer managers across 7 geographic regions to ensure that the survey context was culturally appropriate. Surveys were administered pre-mission (baseline; on the first day) and post-mission (on the last day) to evaluate attitude changes. Questions focused on volunteer demographics, prior exposure to female professionals, and experience with mentorship and leadership. Opinion questions used a 4-point Likert scale.

Pre- and post-mission survey responses were analyzed independently and as paired data. Responses were analyzed according to age, country of origin (geographic region and World Bank income level grouping) **[11]**, country gender equity ranking (World Economic Forum Global Gender Gap Index) [12], mission role, and prior mentorship experiences. Data were recorded in RedCap (Vanderbilt University, Nashville, TN) and analyzed using student’s t-test or chi-squared. Analyses were performed in Excel (Microsoft Corp, Redmond, WA) and R (R Core Team, Vienna, Austria). Statistical significance was defined as p<0.05.

## Results

### Descriptive statistics

**Fig 1:**
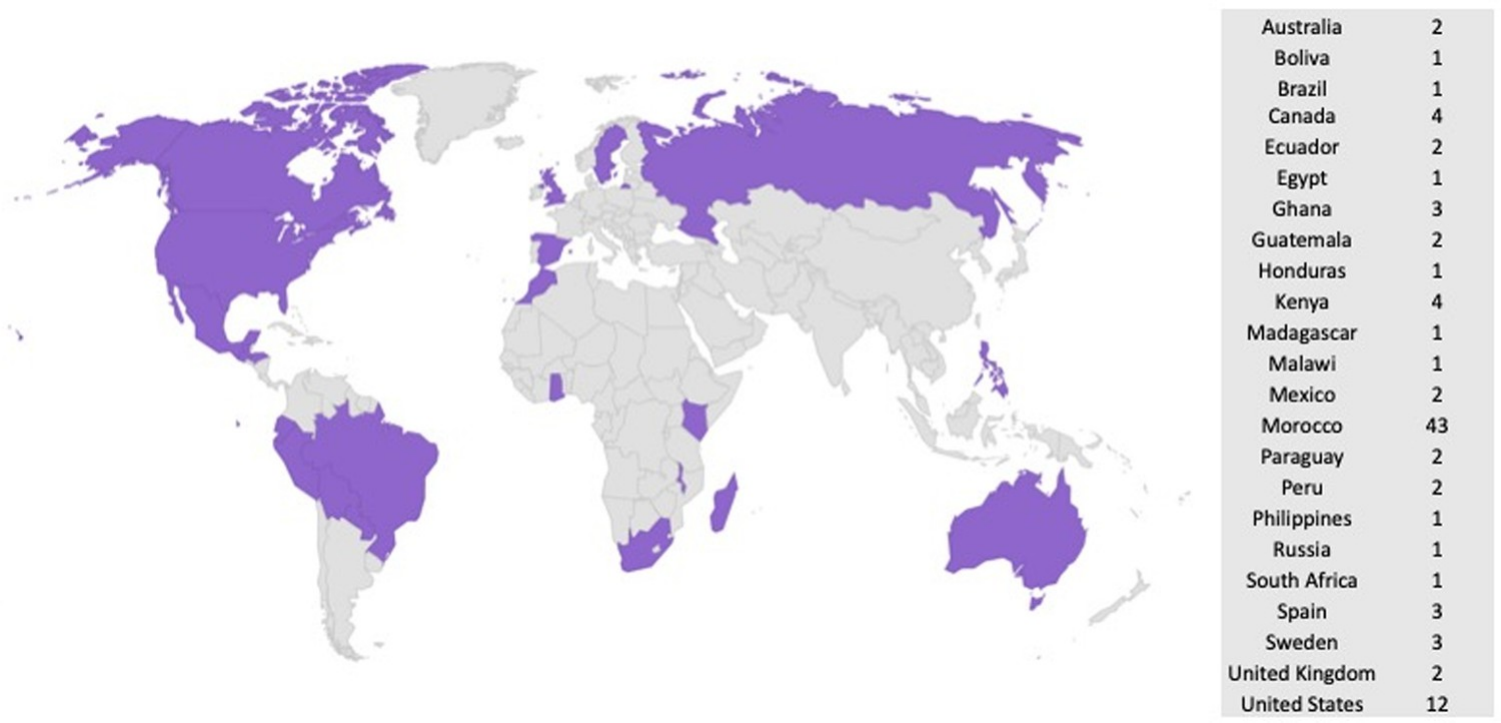
Home countries of volunteers who participated in the Oujda, Morocco mission. This map was generated and published with approval from amCharts (Vilnius, Lithuania).

95 women participated in the mission and all were eligible for the study. 70 (74%) were medical volunteers (physicians, nurses, and other medical professionals) and 25 (26%) were non-medical volunteers (administrators, translators, or students). Volunteers came from 23 different countries and 68% were from LMICs (Fig 1).

Pre-mission, post-mission, and both surveys were completed by 85%, 81%, and 74% of volunteers, respectively. Volunteers had an average of 8.1 ± 7.3 years’ experience working with Operation Smile. The majority of participants had been on 11–15 prior missions with the organization. 29% of volunteers had prior experience as a team leader and 28% as an educator for Operation Smile. Volunteers from HICs and LMICs had similar mission roles (p = 0.58), number of prior missions (p = 0.47), duration of volunteerism (p = 0.69), team leader experience (p = 0.28) and educator experience (p = 0.18) (Table 1).

**Table 1:** Demographics of survey respondents from high income countries (HICs) and lower- and middle-income countries (LMICs).

	HIC (n = 22)	LMIC (n = 56)	Overall (N = 81)	P-value
**Region**				
East Asia and Pacific	2 (9.1%)	1 (1.8%)	3 (3.7%)	
Europe and Central Asia	7 (31.8%)	1 (1.8%)	8 (9.9%)	
Latin America & the Caribbean	1 (4.5%)	11 (19.6%)	12 (14.8%)	
Middle East and North Africa	0 (0%)	30 (53.6%)	31 (38.3%)	
North America	12 (54.5%)	3 (5.4%)	17 (21.0%)	
Sub-Saharan Africa	0 (0%)	10 (17.9%)	10 (12.3%)	
**Age, years**				
Mean (SD)	41.1 (15.1)	40.0 (14.4)	40.5 (14.7)	0.77
Median [Min, Max]	41.5 [17.0, 65.0]	36.0 [20.0, 73.0]	39.0 [17.0, 73. 0]	
**Role on the Mission** †				
Doctor	6 (27.3%)	17 (30.4%)	23 (28.4%)	0.58
Nonmedical	8 (36.4%)	12 (21.4%)	20 (24.7%)	
Nurse	4 (18.2%)	15 (26.8%)	21 (25.9%)	
Other Medical	4 (18.2%)	12 (21.4%)	17 (21.0%)	
**Number of Missions Previously Attended**				
4 or fewer	9 (40.9%)	16 (28.6%)	25 (30.9%)	0.47
5–10	5 (22.7%)	10 (17.9%)	15 (18.5%)	
10–15	4 (18.2%)	8 (14.3%)	14 (17.3%)	
16–20	0 (0%)	5 (8.9%)	5 (6.2%)	
21–25	0 (0%)	3 (5.4%)	3 (3.7%)	
26 or more	4 (18.2%)	14 (25.0%)	19 (23.5%)	
**Duration of Volunteer Engagement, years**				
Mead (SD)	7.7 (7.3)	8.2 (7.4)	8.1 (7.3)	0.69
Median [Min, Max]	5 [0. 27]	6 [0, 25]	5 [0, 27]	
**Prior Operation Smile Roles**				
Team Leader	4 (18.2%)	19 (33.9%)	24 (29.6%)	0.18
Educator	8 (36.4%)	14 (25.0%)	23 (28.4%)	0.28

† Doctor includes anesthesiologists, cleft surgeons, PACU physicians, pediatricians; Nonmedical includes administrators, translators, and students; Nurses includes clinical coordinators, operating room, PACU and ward nurses; Other medical includes dentists, speech language pathologists, child life specialists, medical records, biomedical engineer, and photography technicians. Standard Deviation (SD), post anesthesia care unit (PACU), high income countries (HIC), and low-and middle-income country (LMIC).

### Female representation in home environments

**Fig 2:**
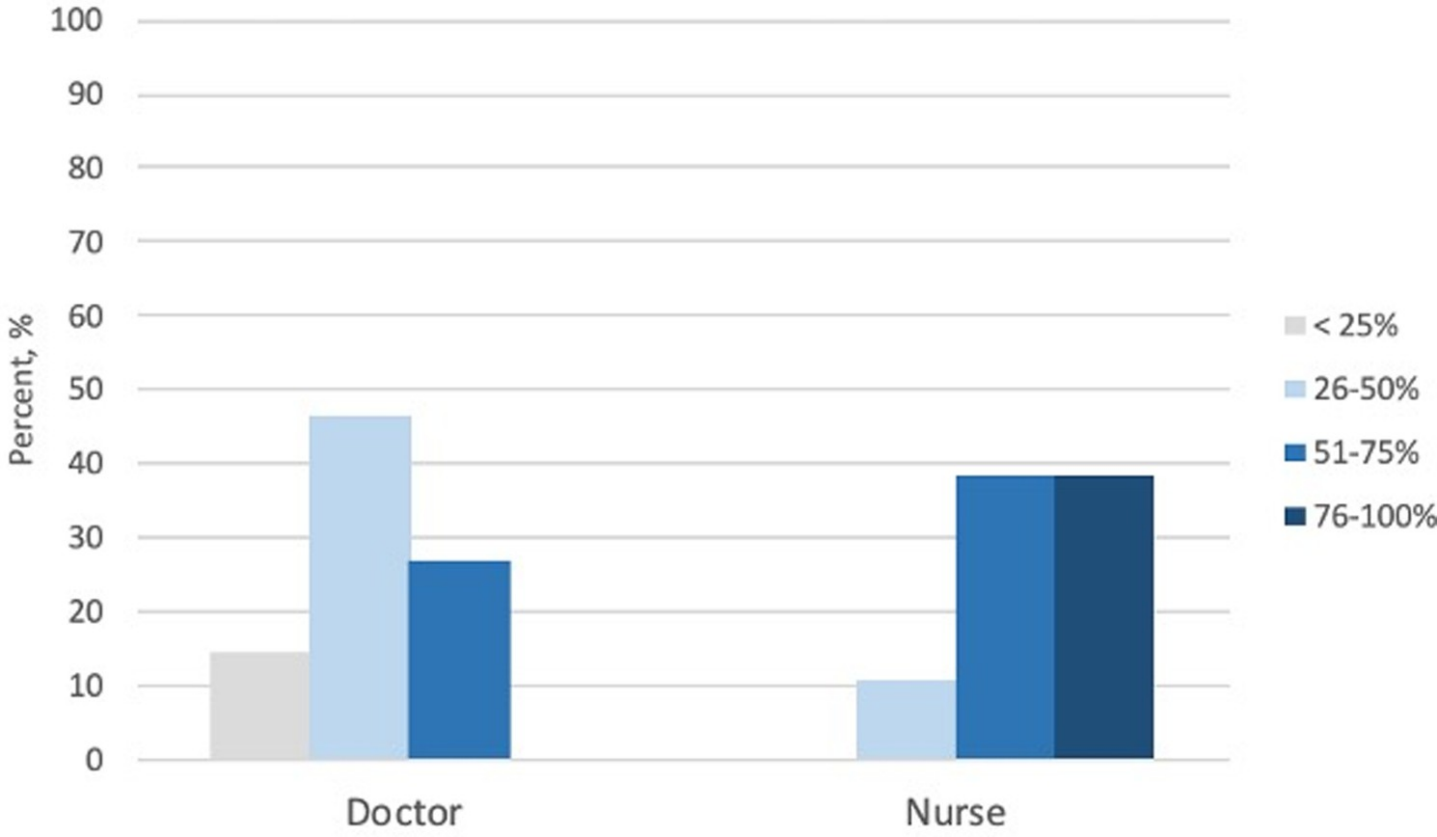
In your home country, what percentage of doctors or nurses are women? volunteer estimates (overall).

**Fig 3:**
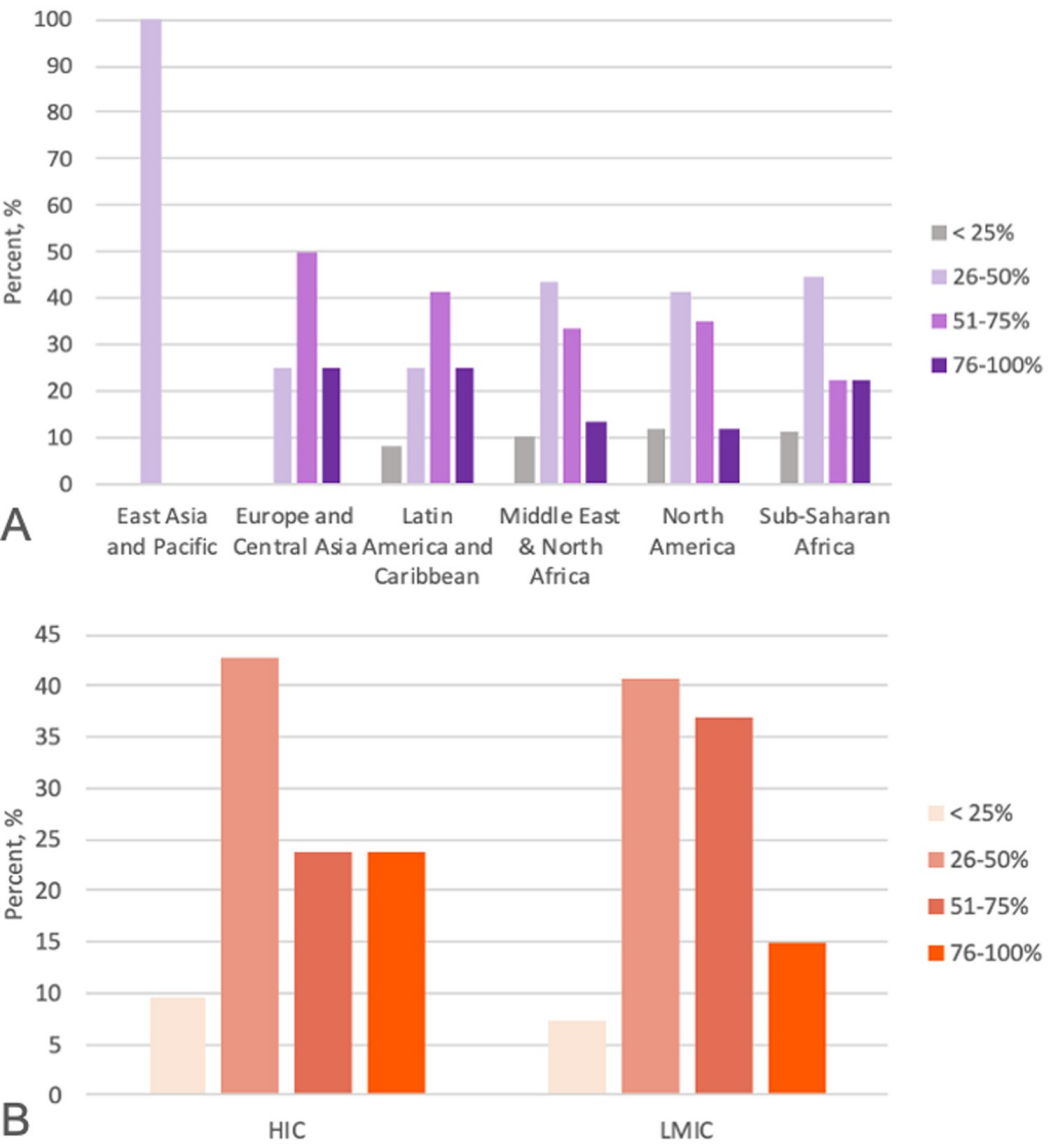
In your home country, what percentage of medical professionals are women? **A:** By volunteer home region. **B:** By volunteer home country income level.

In their home countries, nurses tended to work in female-dominated workplaces whereas physicians tended to work in male-dominated environments (Fig 2). Volunteers from Europe and Latin America worked with more women professionally versus sub-Saharan Africa, the Middle East and North Africa, and North America. Only 3 volunteers originated from East Asia and the Pacific (Fig 3A). Volunteers from LMICs and HICs had similar estimates for prevalence of female healthcare workers at home (p = 0.66) (Fig 3B).

### Mentorship

**Fig 4:**
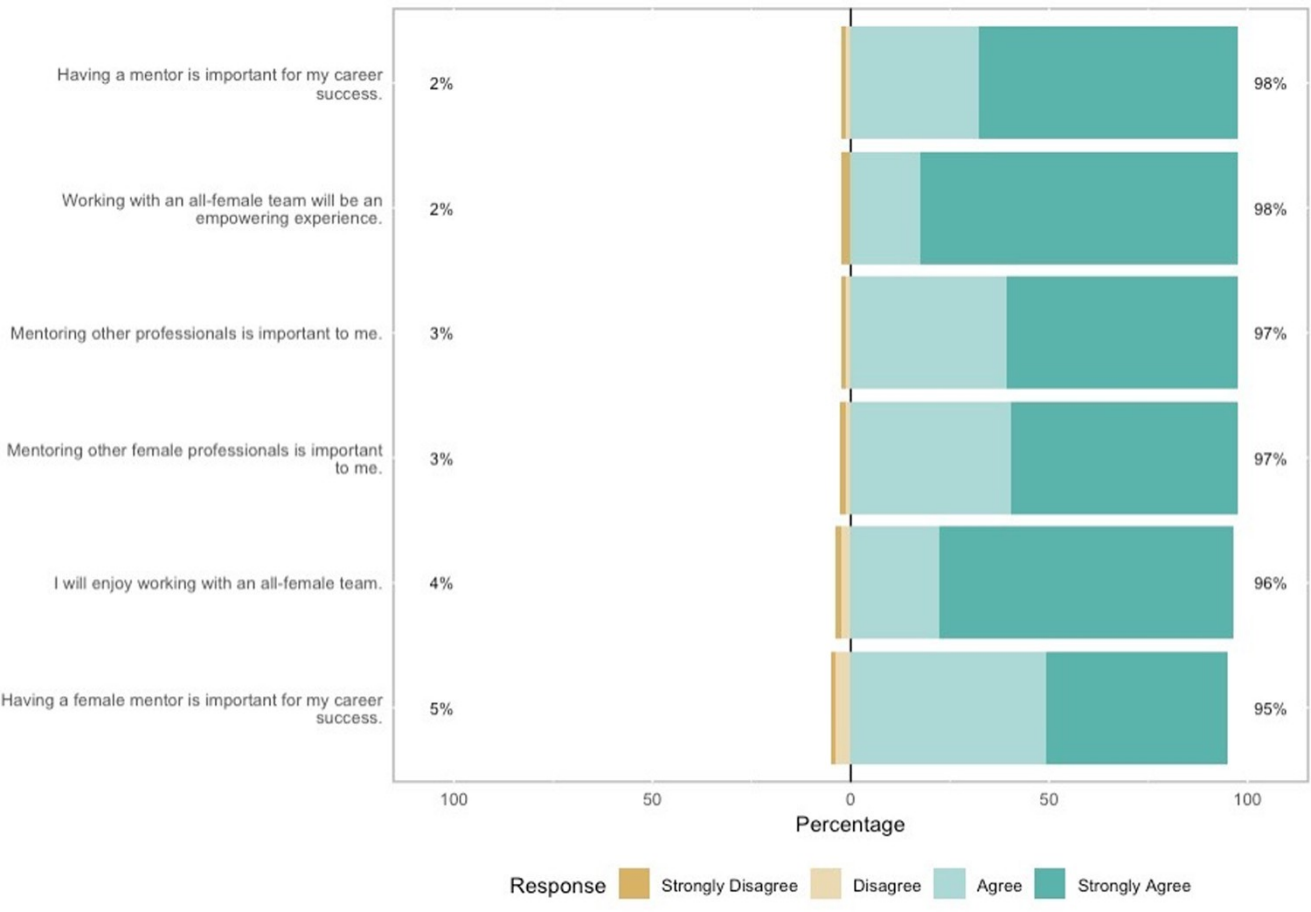
Opinions on mentorship before the mission.

Most women had mentored or received mentorship from another woman before (Table 2). 73% had previously received mentorship from a colleague, most of whom were women (90%). Similarly, 75% of women had previously mentored a colleague; most of their mentees were female (93%). Nearly all volunteers wanted to be mentors for others, especially for women (97% and 97%, respectively) (Fig 4). However, women from both HICs and LMICs struggle to get enough mentorship. 98% of volunteers felt their mentorship was insufficient and 95% preferred a female mentor. Volunteer role (doctor, nurse, other medical, non-medical) was not associated with receiving (p = 0.13) or giving (p = 0.12) mentorship.

**Table 2:** Prior experience with female mentorship.

	HIC (n = 22)	LMIC (n = 56)	Overall (N = 81)	P-value
**Had experience working in an all- female professional environment**	8 (36%)	18 (32%)	26 (32%)	0.91
**Received mentorship from a colleague**	17 (77%)	39 (70%)	59 (73%)	0.97
**Received mentorship from a female colleague**	16 (73%)	34 (61%)	53 (65%)	0.73
**Mentored a colleague**	17 (77%)	41 (73%)	61 (75%)	0.64
**Mentored a female colleague**	15 (68%)	39 (70%)	57 (70%)	0.90

Living in a HIC versus LMIC did not influence prior experience receiving mentorship (77% vs 70%, p = 0.97) or being mentored by a woman (73% vs 61%, p = 0.73). Similarly, giving mentorship to a colleague (77% vs 73%, p = 0.64) or female colleague (68% vs 70%, p = 0.90) was equivalent in HICs and LMICs.

**Fig 5:**
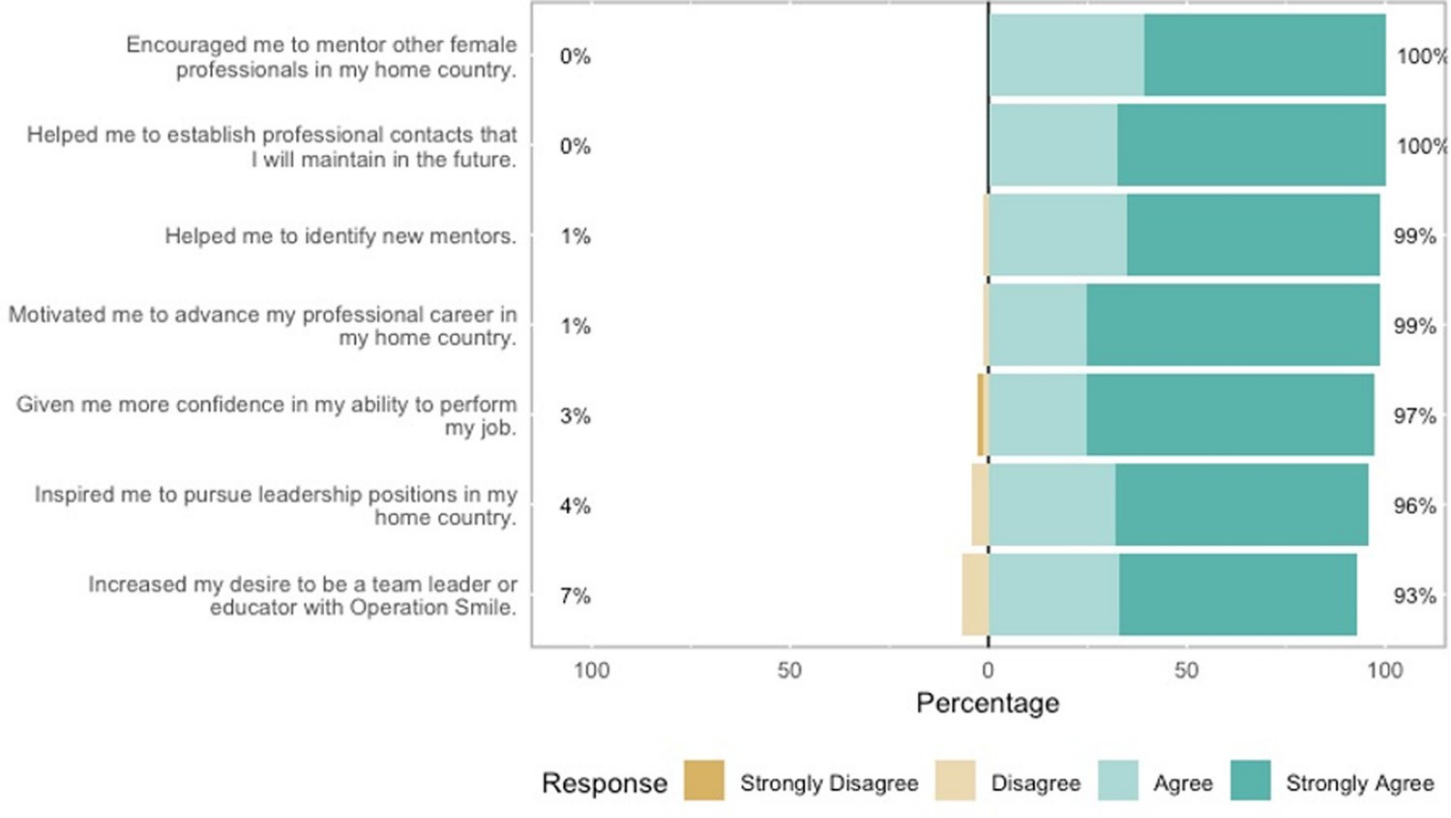
Opinions on mentorship and leadership after the women’s mission.

At this mission, many women gave and received mentorship for the first time. 68% mentored others; 42% who had never mentored before became mentors for the first time. 77% of volunteers received mentorship.15 participants had never been mentored before, and 11 of them (73%) received mentorship for the first time. Mentorship during the mission may have a trickle-down effect for participant home countries. 100% established professional contacts that they planned to maintain in the future and 100% felt empowered to mentor working women at home (Fig 5).

### Experience in prior all-female environments

26 women (32%) had prior experience working in an all-female professional environment. Prior work in an all-female environment was not associated with the volunteer’s profession (p = 0.81), self-reported gender equity at home (p = 0.53), or prior mentorship experience (giving p = 0.93; receiving p = 1.0).

### Leadership development

Participants anticipated being empowered by the all-female mission experience with 98% expecting empowerment before and 99% reporting empowerment afterwards (p = 0.20). Volunteers did not expect to enjoy the mission as much as they did, with 75% expecting to enjoy the experience before versus 87% reporting they enjoyed the experience after (p = 0.04).

**Fig 6:**
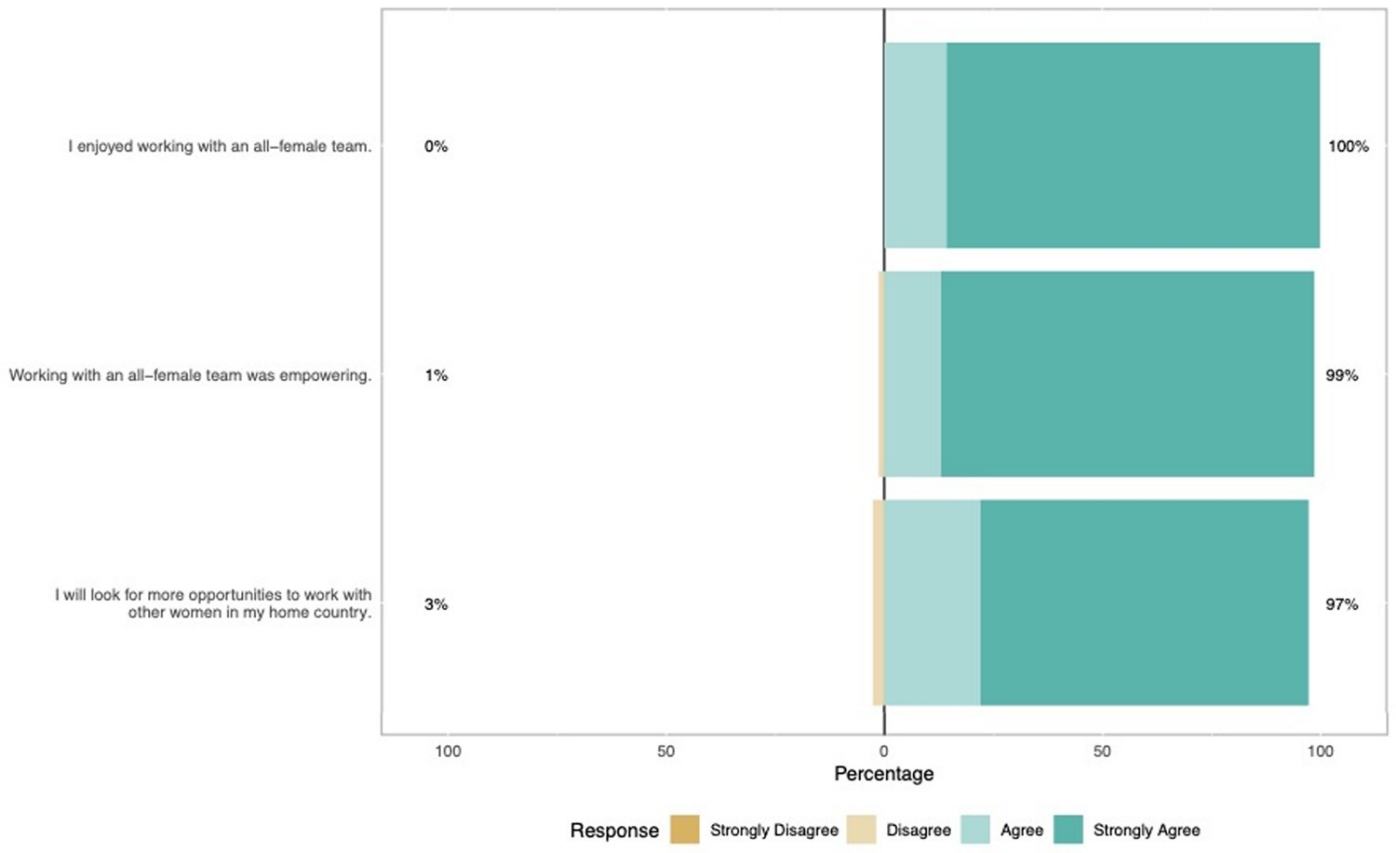
Opinions on working with other women after the women’s mission.

Only 16% of participants were team leaders. Nevertheless 99% felt inspired to pursue leadership positions in their home countries and 93% within Operation Smile. 99% of participants felt motivated to advance professionally in their career and 97% wanted to work with other women in their career (Fig 6).

## Discussion

Overall, study participants reported that the all-female work environment was enjoyable, empowering, and career motivating. The volunteers from HICs and LMICs reported equivalent gender inequality in their home countries. Fewer women were working as doctors versus nurses. These data are consistent with the literature that reports the majority of healthcare workers are women, but fewer hold higher skilled jobs [13].

### Perceptions of gender inequity in medicine may relate to overall country gender equality not country economic standing

According to the World Economic Forum’s Global Gender Gap Index (GGGI), women have less equality in the Middle East and North Africa (MENA), sub-Saharan Africa, and Asia [12]. Our volunteers approximated the prevalence of women in healthcare in their home countries; their estimates suggest countries with lower gender equity rankings may have fewer women than men working in professional environments. Volunteers from Europe and Latin America reported greater presence of female professionals in the workplace, consistent with the regions’ higher gender equity ranking **[12]**. In contrast, North America has a high gender equity rating, but volunteers reported fewer women in healthcare. This disparity exists despite an equivalent percentage of women working in healthcare in Europe and the U.S. **[14, 15]**. Differences between documented reports and volunteer opinions suggest that perception of work environments may be influenced by overall country gender equity in addition to other factors, such as the individual’s work environments and personal perceptions.

### Mentorship appears universally difficult to achieve for women in medicine

Prior to this mission, 25% of women reported not receiving the mentorship they wanted in their home countries, regardless of profession, country economics, or region. Similar to reports from Xepoleas *et al* on the experience of women in healthcare, this finding suggests that lack of mentorship is a struggle for women as a whole rather than a specific cultural, economic, or career phenomenon **[10]**.

The insignificant difference in mentorship needs between women from HICs and LMICs may result from a universal lack of female representation in higher positions within healthcare [3, 4]. Limited number of women in healthcare leadership positions can enforce a glass ceiling, which further restricts other women from advancing to higher positions. The shared desire for mentorship between women in HICs and LMICs may reflect gender-norms structures that are pervasive across cultures, such as women serving as primary caretakers and having to managing childrearing with professional work [16]. Due to this shared experience of women, female healthcare professionals may specifically seek out female mentorship. In a study of female South African surgeons, 22% reported that the gender of their mentor made a difference in the quality of their training **[8]**. Female-to-female mentorship may be able to offer women gender-specific guidance in traditionally male-dominated healthcare fields such as surgery.

According to volunteers, the all-female mission provided a safe space to teach and be taught. Both of these experiences are essential, as mentoring is a learned skill. Studies suggest that mentoring capabilities evolve over time and can lead to the professional development of both the mentor and mentee [17]. Within this group of women, those who were not designated as team leaders on the mission still reported engaging in mentoring roles. A previous small-scale study on female mentorship in medicine corroborate our findings; an overall supportive environment may inspire peer teaching and engagement **[18]**.

The impact of the all-female environment appeared to extend beyond the mission. Participants reported establishing professional contacts they intended to maintain, which may allow for continued mentorship and career development. Individuals reported being inspired to pursue mentorship when returning home. A 2017 study in the U.S. found that access to a mentor-mentee relationship, especially at earlier stages of a career, can lead to greater retention and the continued sustainability of women in science, technology, engineering, and math (STEM) fields [19]. The present study, along with previous literature, showcases that women can educate other women with likely lasting impact.

### The all-female professional environment helped encourage women to become leaders

Globally, pursuing leadership positions can be challenging for women. A study of female oncologists in the Middle East reported that mothers were discouraged from pursuing leadership even though they felt capable [20]. In an expanded study of female healthcare workers in the U.S., Haiti, Tanzania, and India, 53% reported that gender discrimination prevented promotions [21].

Despite challenging environments, participants on the all-women mission reported being inspired to pursue leadership positions at home. Several reasons may have helped inspire leadership growth as a result of this unique experience. As discussed by Mathad *et al*, a key component for women in medicine to advance professionally is to have public support of gender equity in their workplace [21]. During the all-female mission experience, participants may have been encouraged by the women surrounding them and felt empowered by how much they enjoyed the experience. Our data also aligns with previous literature that shows women find it meaningful and enjoyable to work together [22]. Having women in leadership roles also decreases gender discrimination in male-dominated fields [23]. These collective sentiments may help explain why almost all participants wanted to continue working with other women and advance in a leadership role at home. Geographic region or country economic status did not affect this desire, suggesting a universal impact of the experience.

### Limitations

Philanthropic environments are not comparable to normal professional environments. Those who volunteer likely have stronger desires to educate, learn, and grow. Additionally, women who had the opportunity to leave their normal responsibilities for a 10-day mission likely had strong work and familial support; these volunteers may not represent the average woman in their country. Nevertheless, over 70% of participants had substantial experience with Operation Smile, participating in 5 or more missions prior to this experience. The significant response to the mission suggests participants were impacted by the unique, all-female mission environment, as opposed to just a philanthropic experience. Secondly, the small number of participants from each country and region limits our ability to draw generalizable conclusions about individual countries. Similarly, the conclusions drawn here suggest the high potential of positive impact from women’s empowerment initiatives. Future studies with more missions and larger cohorts are needed to reaffirm the conclusions suggested by the data presented. Lastly, longitudinal follow up of this cohort is needed to confirm long-term impact of the mission experience.

### Follow-up

In the future, we plan to study control groups: women who did not participate in a mission, or women who participated in a mixed-gender mission. This future study aims to separate the effects of an all-female workplace from general philanthropic participation.

## Conclusion

Initiatives that facilitate collaboration between women and bring professional women into an all-female work environment, even if through a temporary experience, may generate lasting impact on their lives and careers through encouraging leadership and pairing of mentors with mentees. Increasing interactions between women in healthcare may lead to career advancement in both HICs and LMICs. Without purposeful initiatives to increase female participation in healthcare, gender equity in medicine will not occur for 200 years [5]. More healthcare providers are needed now; if governments and healthcare systems promote women in medicine, the needs of the global population have the potential to be met faster.

## Abbreviations

U.S.: United States of America
HIC: High Income Country
LMIC: Low- and- Middle- Income- Country
